# Determinants of Knowledge, Attitudes, Perceptions and Behaviors Regarding Air Pollution in Schoolchildren in Pristina, Kosovo

**DOI:** 10.3390/children11010128

**Published:** 2024-01-19

**Authors:** Zana Shabani Isenaj, Hanns Moshammer, Merita Berisha, Lisbeth Weitensfelder

**Affiliations:** 1 Medical Faculty, University of Hasan Pristina, George Bush 31, 10000 Pristina, Kosovo; zana.shabani1@student.uni-pr.edu (Z.S.I.); merita.berisha@uni-pr.edu (M.B.); 2 Department of Environmental Health, Zentrum für Public Health, Medical University of Vienna, 1090 Vienna, Austria; lisbeth.weitensfelder@meduniwien.ac.at

**Keywords:** air pollution, air quality, environmental health literacy, knowledge, attitude, perception and behavior

## Abstract

Air pollution poses a significant public health challenge, and Kosovo, a low-middle-income country in the Balkan peninsula, suffers from particularly poor air quality, especially around the area of the capital Pristina. The availability of accurate and timely information is crucial in mitigating the adverse effects of air pollution. This study aimed at evaluating the knowledge, attitudes, behaviors, and perceptions (KAPB) related to poor air quality in Pristina’s low-middle schools. Furthermore, the study explored the connections between these factors and socio-demographic and health attributes and provided valuable inputs for the development of future strategies and policies in air pollution mitigation. Regression analysis provided insights into how these various factors interacted with KAPB scores. The results revealed limited knowledge about air pollution sources and risks among pupils, with insufficient awareness of reliable information sources. While attitudes were generally positive, they declined with higher grade levels. Parental education significantly influenced knowledge and attitudes, and better health correlated with more positive attitudes. Perceptions of air pollution risks were influenced by grade, gender, and parental education, with better-educated parents associated with improved perceptions. Overall behavior scores increased with higher levels of parental education. Understanding the factors that shape pupils’ responses to air pollution is critical for strategy and policy development. These findings can guide strategies to enhance environmental awareness and promote healthy behavior, helping address the pressing issue of air pollution in the country.

## 1. Introduction

Air pollution poses a global health challenge, impacting the well-being of millions worldwide [1]. Exposure, whether short or prolonged, increases the risk of severe health conditions, such as cancer and respiratory and cardiovascular disorders, that contribute to elevated morbidity and mortality in numerous countries [2]. In a recent publication, the World Health Organization (WHO) estimated that the annual global death toll due to exposure to high levels of outdoor air pollutants, including fine particles (PM 2.5), is 4.5 million, with about 89% occurring in developing countries. Vulnerable groups, including the elderly, children, pregnant women, and those with chronic illnesses, are especially prone to the adverse effects of pollution [2,3]. Numerous studies have firmly established a link between air pollution exposure and various illnesses, including respiratory issues in both newborns and adults [4,5], cardiovascular diseases [6,7,8], cancer [9,10], mental health issues, neurocognitive disorders [11,12], and increased mortality [13,14]. The cognitive development of primary school children may be adversely influenced by exposure to air pollutants generated by traffic, potentially leading to diminished learning skills. A study by Sunyer et al. (2015) found that children attending schools with high pollution levels experienced slower growth in cognitive development compared to those in schools with lower levels of air pollutants [15]. Margolis et al. (2021) discovered that increased prenatal exposure to PAH was linked to poorer inhibitory control in late childhood and diminished academic skills in early adolescence. Additionally, investigations into the prolonged effects of air pollution have quantified its negative impacts on respiratory health, mental well-being, and early-life mortality [16,17,18]. Kim et al.’s (2017) research revealed a substantial impact on depression and depressive-like symptoms, which persisted for up to ten years after pollution exposure [16]. Another longitudinal study by Jayachandran [18] demonstrated that long-term exposure to severe pollution significantly affected fetal, infant, and child mortality. Furthermore, a study by Kim and Radoias (2022) reported reduced lung capacity two decades after exposure to severe pollution [19].

The impact of air pollution is particularly pronounced in low- and middle-income countries, and Kosovo, a small Balkan nation, exemplifies this concern with heightened pollution levels, especially during the winter season [20]. As per a recent report from the World Bank, about 760 individuals in Kosovo experience premature deaths annually due to ambient air pollution. The city of Pristina, being both the most populated and polluted, contributes to 11% of these cases among its over 200,000 residents in 2021. Among the pollution-related fatalities, 90% are attributed to a combination of ischemic heart disease and stroke [20]. A study conducted by Isenaj et al. in 2022 revealed that short-term exposure to air pollution in Pristina was linked to an increased number of hospital visits by children due to respiratory issues [21]. The primary factors contributing to this situation include the combustion of solid fuels in homes, outdated large thermal power plants, industrial emissions, and vehicle exhaust. Efforts to reduce air pollution-related health problems can be effective through strategies implemented at both the individual and community levels [22]. The effective formulation of strategies relies on grasping the knowledge, attitudes, and practices linked to air pollution at both the individual and community levels [22]. The understanding and response of people to outdoor air pollution are pivotal for protecting the health of urban residents. Recent studies show that individuals are conscious of the adverse effects of air pollution and are willing to invest in actions to improve air quality [23]. Increasing awareness can significantly influence behavioral changes, reducing personal exposure to air pollution and individual emissions. This transformation contributes to enhanced air quality and promotes the adoption of eco-friendly transportation methods, such as walking, cycling, and public transport [24]. Given the potential long-term impacts of environmental mistreatment on quality of life, research has predominantly focused on understanding the environmental knowledge, attitudes, and behaviors of college and high school students. However, the existing body of research on elementary school children’s environmental awareness is limited [25]. For instance, Loughlan et al.’s 2022 study on young people’s perceptions of the environment underscores the significance of environmental education in schools as a crucial strategy for environmental enhancement. However, this education should be grounded in children’s actual understanding of the environment rather than relying on assumptions about what children know and believe [26]. Tuncer et al. (2005) investigated the environmental attitudes of young people in Turkey and explored the influence of school type and gender [27], with females exhibiting more positive attitudes toward the environment and a greater awareness of individual responsibilities and overall environmental problems than boys. Makki et al. (2003) reported that the educational attainment of a parent influenced the knowledge and understanding of the environment among students [28]. In a study conducted by Boell De Paw et al. in 2012, the emphasis was on cultural variations in the environmental perspectives of children. The findings highlighted a distinct and notably substantial cultural impact on children’s environmental outlook. These disparities hold significance for those involved in designing and assessing environmental education initiatives, emphasizing the necessity for initiatives to be contextually grounded in the specific local situation [29]. Our study seeks to fill the wide gaps in the literature specifically by assessing the level of knowledge, attitudes, behaviors, and perceptions in low-middle school children and their associations with socio-demographic factors in Pristina, Kosovo. The aim is to gather baseline data for a more extensive environmental intervention study. This larger study, stemming from the initial research, will evaluate the impact of an educational intervention on students’ knowledge, attitudes, behaviors, and perceptions concerning outdoor air pollution. Through this investigation, we seek to gain a deeper understanding of the factors that influence pupils’ understanding and responses to air pollution, with the ultimate goal of informing strategies and policies aimed at addressing this critical environmental issue in the Pristina region.

This study is a baseline survey that will be followed by an intervention: In half of the schools, lectures on air pollution will be offered, while the remaining schools will serve as controls. In a second survey round using the same instrument, the effect of the lectures on knowledge and attitudes will be assessed using comparisons between the intervention and control schools and between the first and the second survey. This analysis of the first survey, in addition to offering valuable information on predictors of knowledge and attitudes, also serves to test the instrument for future improvement.

## 2. Materials and Methods

### 2.1. Study Design and Recruitment of Study Subjects

This is a cross-sectional study, and data were collected from a sample of 2560 participants from 8 low-middle schools in Pristina, the capital city of Kosovo. We employed a multi-phase random sampling technique to recruit the participants. From 42 primary schools in the municipality of Pristina, of which 24 are urban and 18 rural, 8 schools have been selected randomly. The study focused on the 6th, 7th, 8th, and 9th grades. We used a standard formula, n = Z^2^ P (1 − P)/e^2^, to reach a representative sample size. In this context, n represents the sample size, p is the baseline level of indicators (0.5), Z reflects the value from the normal distribution associated with the 95% confidence level (1.96), and e stands for the margin of error. We assumed a 0.5 baseline level due to a lack of previous data in the study population. However, this number was adjusted to account for the design effect of the sample design (1.5), the number of age–sex estimates (4 age–sex estimates) to be reported, and the anticipated non-response (20%); the preferred sample size reached 2880 participants.

### 2.2. Data Collection and Questions Asked

This study was carried out in 2023 in eight low-middle schools from the municipality of Pristina, covering urban and rural areas. Subjects were pupils from the 5th to the 9th grades. The response rate was 88.8 percent (2560 out of 2880).

Initially, a tool that encompasses inquiries on air pollution sources, health effects, protective measures, attitudes, perceptions, and behavior was created. The questions were developed through a review and adaptation of items from previous studies [22,30,31,32,33,34,35]. The instrument underwent validation by a group of public health experts and was tested in three schools before its finalization. The survey pilot yielded significant results, which were integrated and adjusted in the final survey version. The questionnaire consisted of 5 parts. The first part asked about the grade and the gender of the respondent, the level of education of the father and the mother (no school (coded 0) up to university (coded 4)), and the health status of the respondent. This last topic included a question on the general health status (poor (coded 1) to very good (coded 4)), a question about the existence of a chronic disease (0/1), and questions on specific diseases (lung disease, heart disease, asthma, cancer, and chronic bronchitis). The second part consisted of 13 knowledge questions (Table A1), which were coded −1 for the wrong answer and 1 for the correct answer. In the knowledge part, pupils had to rate statements (e.g., regarding air pollution sources in the region or regarding vulnerable groups) as true or false. “Do not know” was coded 0. The third part consisted of 4 questions on attitudes (Table A2) ranging from “completely agree” to “completely disagree”, which were coded from 1 to 5. The attitude questions contained statements and opinions ranging from traffic jams to how others should behave. The fourth part consisted of 7 questions on risk perception (Table A3). Risk perception included topics like pollution-caused health concerns or perception of air pollution, with possible answers ranging from “never” or “not at all” or “do not agree” until “every day” or “very” or “completely agree”, coded from 1 to 4, 1 to 5 or 1 to 6, respectively. The fifth part consisted of 3 questions on practical behavior (public transport, energy consumption, and reduction in outdoor activities; Table A4) with possible answers ranging from “never” to “yes, always” (coded 0–3).

### 2.3. Data Analysis

The data were imported into a spreadsheet and analyzed using STATA Version 17 [36]. Summary scores were produced from the last four categories of answers. Thus, the sum of the 13 knowledge answers ranged between −13 (if all answers were incorrect) and 13 (if all were correct). The sums of the 4 attitudes questions took on values between 4 and 16, and so on. As the scores were equally spaced and fairly normally distributed, multiple linear regressions were run with each score as the dependent variable. The responses to the first part of the questionnaire were used as independent variables: grade and gender, father’s educational achievement, and general health status. As the educational achievements of both parents were assumed to be highly correlated, it was not reasonable to include both in the same model. The same was the case for the health variables. The models were simplified using the stepwise exclusion of insignificant variables (*p* > 0.1; point estimates of the other variables were not changed by more than 10% upon removal). If the father’s educational achievements turned out to predict a summary score significantly, it was replaced by the mother’s educational achievement to determine which “education” variable provided the better fit. When the health status was significant, we tested whether this was also true for any one disease.

When a summary score was found to be predicted by some of the independent variables, it was also tested to determine which of the answers in that summary category drove the association. This last analysis was not hypothesis-driven, and therefore, our aim was not to generate evidence. Rather, it was meant to test the validity of the single questions as part of the summary score. To that end, the knowledge questions were recoded: “Do not know” was recoded as missing, and the wrong answers were coded as 0. Thus, a simple logistic regression could be performed on each question separately. For an easier comparison with the results of the linear regression, the results of the logistic regressions were also presented as coefficients or log odds. For the answers of the three other response categories, ordered logistic regressions were performed, and coefficients were also reported. Moreover, 95% confidence intervals (CIs) were presented, and *p* < 0.05 was considered statistically significant. The other responses (attitudes, perceptions, and behaviors) were analyzed using ordered logistic regression.

### 2.4. Ethics Approval

The University of Hasan Pristina’s Faculty of Medicine’s Research Ethics Committee granted ethics approval for this study. Written letters of interest and confirmation were obtained from each school and the Education Directorate of the Municipality of Pristina. A verbal agreement was accepted as consent to participate in the survey. Participants were briefed that the survey was anonymous and were informed that they had the right to withdraw at any point.

## 3. Results

### 3.1. Descriptive Statistics

The educational achievements of the fathers and the mothers (Table 1) were highly correlated with each other (Spearman’s rho = 0.628, *p* < 0.001), and so were the health indicators (Table 2): pupils without a disease reported a 0.28 point better overall health status than those reporting a disease (*p* < 0.001), and girls (51%) and older children (13–15 years, 56.2%) dominated somewhat over boys and younger children (10–12 years).

**Figure 1:**
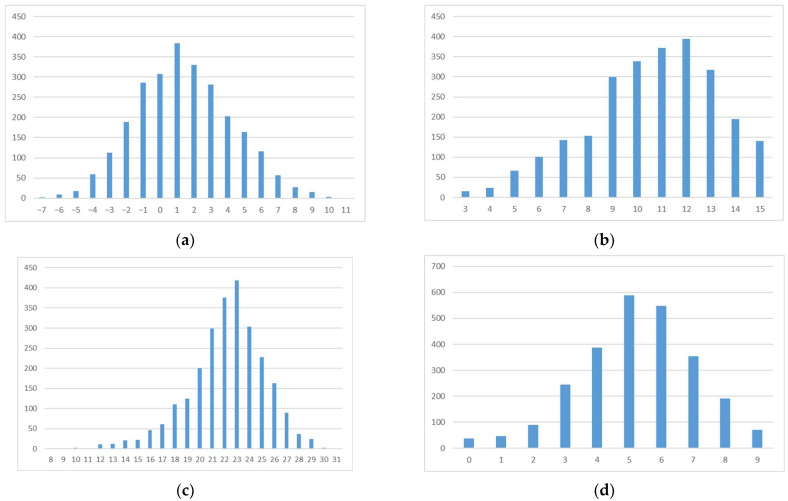
Distributional plots of the scores for (**a**) knowledge; (**b**) attitudes; (**c**) perceptions; and (**d**) behaviors.

The summary scores for knowledge, attitudes, perceptions, and behaviors each sufficiently approximated a normal distribution (Figure 1), allowing for linear regression analysis.

### 3.2. Regression Analyses

Regarding all regression models and all investigated dependent variables, explained variance (R-square values) rarely exceeded 0.02.

**Figure 2:**
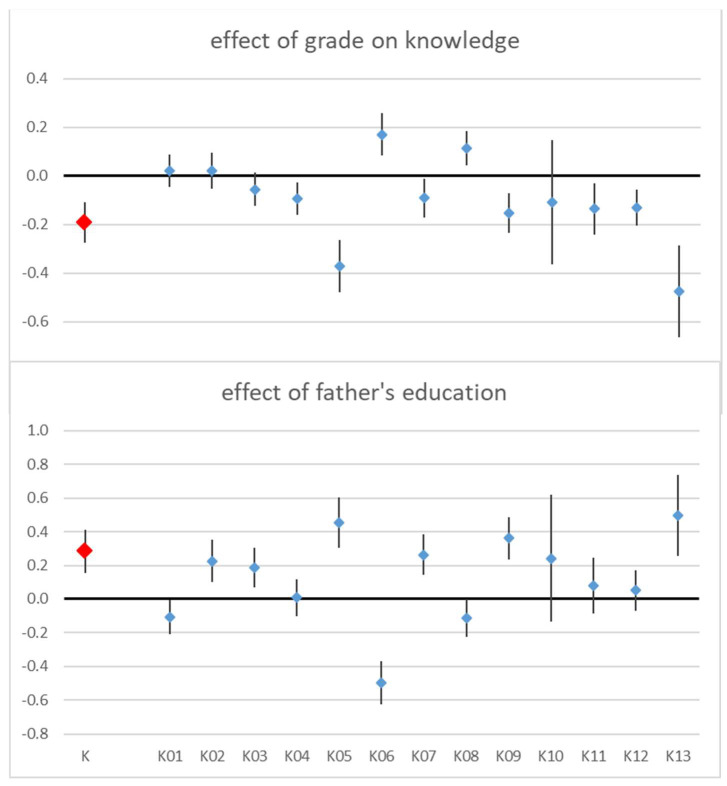
Results of linear regression on knowledge score (K, in red) and of logistic regressions on the response to each knowledge question (K01–K13, in blue), coefficients and 95% conf. intervals.

The knowledge score was affected by grade and by the father’s education. Interestingly, the higher the grade, the poorer the overall knowledge (coefficient = −0.19, *p* < 0.001). The father’s education was a little more predictive (t = 4.33) than the mother’s education (t = 3.7), and overall knowledge increased with the parents’ educational achievement (father’s: coefficient = 0.28, *p* < 0.001). When both parents’ education was included in the model, only the father’s education remained significant. Not all knowledge questions contributed equally to this result (Table 3, Figure 2).

**Figure 3:**
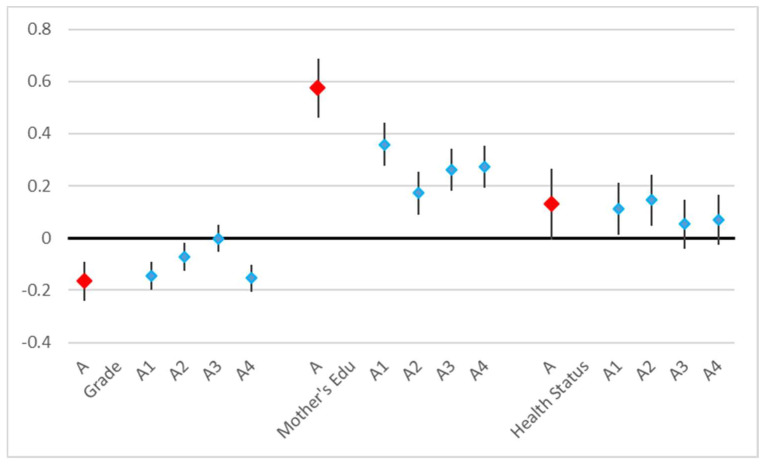
Results of linear regression on the attitudes score (A) and of ordered logistic regressions on the response to each knowledge question (A1–A4), coefficients, and 95% confidence intervals.

The attitudes score was affected by the grade, the mother’s education, and the health status. Again, the higher the grade, the poorer the overall attitude (coefficient = −0.17, *p* < 0.001). The mother’s education was a little more predictive (t = 9.92) than the father’s education (t = 8.27), and the overall attitude increased with the parents’ educational achievement (mother’s: coefficient = 0.57, *p* < 0.001). Only the mother’s education remained significant when both educations were included in the model. A better health status predicted better attitudes (coefficient = 0.13, *p* = 0.05). All attitudes questions contributed similarly to this result (Table 4, Figure 3). Among specific diseases, only reported lung disease (coefficient = −0.64) contributed significantly (*p* = 0.037) and negatively to overall attitudes.

**Figure 4:**
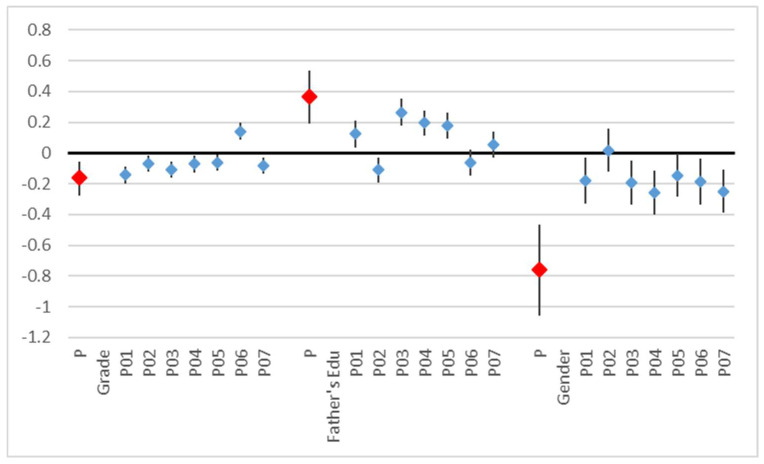
Results of linear regression on the perceptions score (P) and of ordered logistic regressions on the response to each perception question (P01–P07), coefficients, and 95% confidence intervals.

The perception score was affected by grade and gender and by the father’s education. Here also, the higher the grade, the poorer the overall perception (coefficient = −0.09, *p* = 0.053). The mother’s education was less predictive (t = 2.15) than the father’s education (t = 4.09) and was no longer significant when both variables were included. Males had a lower perception overall (coefficient = −0.28, *p* = 0.019). Not all perception questions contributed equally to this result (Table 5, Figure 4).

**Figure 5:**
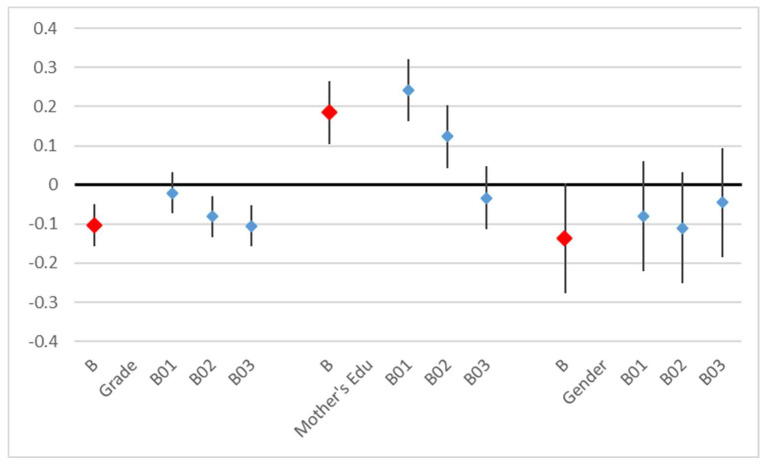
Results of linear regression on the behavior score (B) and of ordered logistic regressions on the response to each behavior question (B01–B03), coefficients, and 95% confidence intervals.

The behavior score was affected by the grade and by the mother’s education. The higher the grade, the poorer the overall good behavior (coefficient = −0.1, *p* < 0.001). The mother’s education was a little more predictive (t = 5.92) than the father’s education (t = 4.5) and was no longer significant when both variables were included; the overall good behavior increased with the parents’ educational achievement. Not all behavior questions contributed equally to this result (Table 6, Figure 5).

## 4. Discussion

Air pollution remains a significant environmental challenge in Kosovo, primarily driven by the combustion of solid fuels in households, outdated large thermal power plants, industrial activities, and vehicular exhaust emissions. This issue particularly impacts the quality of air in the urban districts of Kosovo. Prolonged exposure to air pollution has been correlated with respiratory and cardiovascular diseases in numerous polluted regions, including Kosovo [21,37,38,39].

The current study delved into the assessment of pupil’s knowledge, attitudes, behaviors, and (risk) perceptions concerning air pollution, examining their connections with social, demographic, and health factors in low-middle schools located in Pristina, Kosovo. Notably, this investigation represents the first of its kind in Kosovo and the broader region, focusing on gauging the knowledge of air pollution, its health implications, and protective measures among young individuals. Our study offers valuable insights into the level of awareness about air pollution’s health risks and contributes to scientific research. It is an important foundation for a larger upcoming interventional study that will assess the impact of an educational intervention on these variables. These results are critical in the context of Pristina, a region grappling with air pollution concerns. By delving deeper into the factors influencing pupils’ understanding and responses to air pollution, we aim to provide a solid basis for the development of targeted strategies and policies to mitigate the effects of air pollution and enhance environmental awareness among the youth in Pristina. Additionally, it supplies crucial theoretical foundations that could enable policymakers to address issues of risk management and communication related to air pollution and implement effective preventive measures focusing on the younger generations. Understanding the public’s perceptions of air pollution and its associated health risks when these individuals are young is vital for crafting policies and interventional programs [40]. These insights are also indispensable for reducing air pollution and mitigating health risks [41].

When it came to knowledge (Table A1) about air pollution, its sources, and the associated risks, respondents displayed a higher awareness regarding the risk of premature death and susceptibility among vulnerable groups (61.1%). However, 68.0% of the participants exhibited a limited understanding of the sources of and contributors to air pollution in Kosovo. Moreover, over half of the respondents were unaware of where to access reliable information about air quality (65.8%). Hence, our findings show the necessity to bring knowledge about the matter to pupils, thereby covering both facts as well as means of empowerment (such as where to find reliable sources of further information).

Regarding their attitudes (Table A2), more than half of the respondents acknowledged air pollution as a serious concern that should receive attention from society (66%), even though their understanding of the matter has been described as limited. However, worries or concerns are not necessarily bound to topics people are well informed about. A significant majority also believed that individuals should take steps to reduce their contributions to air pollution (71.5%).

The study also assessed the perceptions of the respondents (Table A3) regarding air pollution, showing that a majority considered air pollution as troublesome for their health. A total of 83.5% agreed that air pollution is perilous to their health, with over 50% expressing concerns about air pollution stemming from industrial activities (62.5%) and transportation (60.6%). Regarding domestic heating, only 42.7% reported concerns. Furthermore, 83.6% reported experiencing difficulties in breathing over the past month.

The present study also inquired about specific behaviors (Table A4) aimed at reducing exposure to air pollution. Using public transport, walking, or cycling was reported by 59.3% of the respondents, while 68.8% claimed to save energy by turning off lights and air conditioning. Additionally, 56.1% stated that they reduce outdoor activities as part of their efforts to mitigate air pollution.

The analysis of parental education revealed a significant positive correlation between the educational achievements of fathers and mothers, with a Spearman’s rho coefficient of 0.628 (*p* < 0.001). This correlation highlights the interdependence of educational attainment within families, indicating that when one parent had a higher level of education, the likelihood of the other parent also having a higher level of education was notably elevated. We assumed that in most households in Kosovo, the income or socio-economic status would be more strongly affected by the educational achievements of the father, while the mother would usually bear the majority of the caregiving and education of the children. Hence, we wondered whether we could discern between “knowledge” of the child (that we assumed would be more strongly influenced by the mother’s educational status) and their “socio-economic status” (more affected by the father’s education) as predictors of the answers. However, the strong correlation between the two limited our ability to do so.

Turning our attention to the health indicators, this study demonstrated that pupils without any reported diseases exhibited an overall health status that was, on average, 0.28 points higher than those who reported having a disease. This finding underscores the significance of health conditions in influencing the general health status of pupils in the study population. However, it remains to be seen which diseases drove the impact of the general health status on attitudes.

The results of our regression analyses shed light on the complex interplay between various factors and the scores related to knowledge, attitudes, (risk) perceptions, and behaviors among the study participants. We observed several noteworthy patterns and associations, though it has to be mentioned that the overall amount of explained variance remained very modest (R-square values rarely exceeded 0.02).

Knowledge, as assessed by the knowledge score, was influenced by both the students’ grade level and the educational attainment of their fathers. Intriguingly, we found an inverse relationship between grade level and overall knowledge, with higher-grade students exhibiting poorer knowledge. Several reasons for that could be possible, one of them possibly being higher skepticism regarding specific knowledge questions in older, but not younger, students, which could have interfered with their actual knowledge. However, to check for such mechanisms, analyses that would go far beyond the previous research would be necessary (e.g., item response theory analyses investigating whether the knowledge questions actually all assess the same dimension or whether they are fair regarding different age groups). Another reason for these grade differences might stem from the problem that many of the questions only contain three answer categories (true, false, I don’t know), which leads to a high a priori guessing probability. Therefore, we also checked for a possible age dependence of the rate of “don’t know” responses. As it turned out, grade had little influence on the frequency of “don’t knows”, and for the three knowledge questions for which this response was significantly affected by grade, the direction of the effect was positive once (speed limits) and negative twice (main cause of air pollution, air quality information). The frequency of “don’t knows” was mostly influenced by the father’s education, with children from a better-off household answering with less hesitation. Some knowledge questions were answered by girls with more confidence. Previous studies also revealed grade-dependent differences in the level of knowledge of air pollution [42]. However, in that study, pupils in higher grades showed higher knowledge scores. Additionally, the father’s education level had a slightly stronger predictive power on knowledge than the mother’s education, and knowledge scores increased with higher levels of parental educational achievement.

When examining attitudes, we found that grade level, the mother’s education, and the health status of the students played significant roles. Similar to knowledge, a negative relationship between grade level and overall attitudes was evident. A previous study by Alp et al. (2006) [42] found a significant correlation among attitudes and grade level, reporting a decline in positive attitudes with grade level. A decline in positive attitudes indeed agrees with literature findings from another environmental topic, namely climate change: a review about youth perception of climate change concluded that in some studies younger children express more worry and are more willing to take action than older adolescents [43]. One of the reasons could stem from an “adolescent dip” that has been shown in sustainability consciousness [44]. Hence, it is plausible that similar mechanisms apply to the topic of air pollution.

The mother’s education demonstrated a slightly stronger influence on reported attitudes towards air pollution than the father’s education. Generally, attitudes improved with higher levels of parental educational attainment, particularly with the mother’s education. Reports in the literature related to the effect of education level on the knowledge, attitudes, and perceptions scores are consistent with our findings that the level of education is significantly associated with our dependent variables [45,46]. A better health status also predicted more positive attitudes. Previous literature findings also reported that air quality perception is influenced by health condition [45,47].

The (risk) perception score was influenced by grade level, gender, and the mother’s education. Once again, a negative association between higher grade levels and poorer overall (risk) perceptions was observed. The mother’s education exhibited a slightly stronger predictive power than the father’s education, and overall perceptions improved with higher levels of parental educational attainment, especially with the mother’s education.

In the case of reported behavior, the behavior score was influenced by the students’ grade level and the mother’s education. Males had a lower perception overall than females. Previous studies also revealed gender-dependent differences in the level of perceptions of air pollution [45,46,47,48,49]. Similarly, regarding knowledge and attitudes, we found a negative relationship between grade level and reported overall pollution-relevant behavior, with higher-grade students exhibiting poorer behavior. The mother’s education had a slightly stronger predictive power than the father’s education, and overall behavior scores increased with higher levels of parental educational achievement, particularly with the mother’s education. A study by Wells et al. found that individuals suffering from respiratory diseases demonstrated a higher likelihood of reporting alterations in their outdoor activities in response to poor air quality [50].

These findings underscore the multifaceted nature of the factors that influence knowledge, attitudes, (risk) perceptions, and behaviors related to air pollution among pupils. Understanding these relationships is essential for developing targeted interventions and educational strategies to enhance environmental awareness and promote healthier behaviors among the younger population.

Methodologically, limitations might include the fact that all calculations were run as univariate analyses in which the interaction affects could not be considered. It is very likely, though, that our three main dependent variables (attitudes, knowledge, and perceptions) are related and affect each other. Research about the interaction mechanisms of these variables should be encouraged.

The applicability of this study to children of various age groups might be constrained due to the specific age range of the participants recruited, who were all in grades 5 to 9 at low-middle schools. This limitation arises from the practical necessity that self-report questionnaires demand that children be capable of reading and assessing their own perceptions. Therefore, this study restricted its participant age range, and consequently, the findings may not extend to younger age categories.

## 5. Conclusions

This study found some predictors of knowledge, attitudes, and behaviors in children regarding air pollution. In the future, an intervention will be implemented, in which half of the schools in the study will receive specific teaching packages about air pollution and the other half will serve as the control group. In the next step, the survey will be repeated to check the effectiveness of the intervention. Because this analysis has indicated that some questions are not sufficiently predictive of the various scores, another analysis will be performed to improve the sensitivity of the instrument.

## Figures and Tables

**Table 1:** Parental education.

Highest Educational Achievement	Fathers	Mothers
No school	129 (5.1%)	129 (5.1%)
Primary school	267 (10.4%)	329 (12.8%)
High school	817 (31.9%)	719 (28.1%)
University	1347 (52.6%)	1383 (54.0%)

**Table 2:** Respondents’ health status and grade.

Health Status	Girls (1306)	Boys (1254)
Poor	27 (2.1%)	18 (1.4%)
Fair	122 (9.3%)	121 (9.7%)
Good	491 (37.6%)	449 (35.8%)
Very good	666 (51.0%)	666 (53.1%)
Chronic lung disease	42	29
Heart disease	8	18
Asthma	23	19
Cancer	10	4
Chronic bronchitis	49	37
5th Grade	154	169
6th Grade	252	246
7th Grade	295	267
8th Grade	316	272
9th Grade	289	300

**Table 3:** Coefficients of the independent variables predicting knowledge.

Independent Variable	First Model: Coefficient, SE ^1^ (p)	Second Model: Coefficient, SE ^1^ (p)
Gender	0.0737366, 0.112 (0.509)	-
Grade	−0.1875547, 0.042 (<0.001)	−0.1915003, 0.042 (<0.001)
Father’s education	0.2782824, 0.066 (<0.001)	0.2832445, 0.065 (<0.001)
Health status	0.1115416, 0.077 (0.146)	-

^1^ SE = Standard error, rounded. R^2^ of first model: 0.0153; R^2^ of the second model: 0.0143.

**Table 4:** Coefficients of the independent variables predicting attitudes.

Independent Variable	First Model: Coefficient, SE ^1^ (*p*)	Second Model: Coefficient, SE ^1^ (*p*)
Gender	−0.1223249, 0.096 (0.201)	-
Grade	−0.206923, 0.036 (<0.001)	−0.2065984, 0.036 (<0.001)
Father’s education	0.2029615, 0.056 (<0.001)	0.2043657, 0.056 (<0.001)
Health status	0.1409589, 0.066 (0.032)	0.1391404, 0.066 (0.034)
Mother’s education		0.2490451, 0.055 (<0.001)
Lung disease		−0.7778208, 0.290 (0.007)
Asthma		−0.8971602, 0.376 (0.017)

^1^ SE = Standard error, rounded. R^2^ of first model: 0.020; R^2^ of the second model: 0.0194.

**Table 5:** Coefficients of the independent variables predicting perception.

Independent Variable	First Model: Coefficient, SE ^1^ (*p*)	Second Model: Coefficient, SE ^1^ (*p*)
Gender	−0.7640997, 0.150 (<0.001)	−0.7617949, 0.150 (<0.001)
Grade	−0.1642967, 0.056 (0.004)	−0.1667237, 0.056 (0.003)
Father’s education	0.360444, 0.088 (<0.001)	0.3642402, 0.088 (<0.001)
Health status	0.0731216, 0.103 (0.478)	-

^1^ SE = Standard error, rounded. R^2^ of first model: 0.0196; R^2^ of the second model: 0.0195.

**Table 6:** Coefficients of the independent variables predicting behavior.

Independent Variable	First Model: Coefficient, SE ^1^ (*p*)	Second Model: Coefficient, SE ^1^ (*p*)
Gender	−0.1340524, 0.072 (0.063)	−0.1335888, 0.072 (0.064)
Grade	−0.1013301, 0.027 (<0.001)	−0.1018183, 0.027 (<0.001)
Father’s education	0. 1563622, 0.042 (<0.001)	0. 1571257, 0.042 (<0.001)
Health status	0.0147107, 0.049 (0.766)	-
Mother’s education		0.1846422 (<0.001)

^1^ SE = Standard error, rounded. R^2^ of first model: 0.0115; R^2^ of the second model: 0.0115.

## Data Availability

The aggregate data presented in this study are available on request from the corresponding author. The individual data are not publicly available due to privacy.

## Appendix A

**Table A1:** Responses to knowledge questions.

Question Topics	Correct	Wrong	Don’t Know
Main cause of air pollution in the Pristina region	670	1740	150
Natural phenomena as a contributor to air pollution	559	1209	792
PM_2.5_ and lung penetration	646	1504	410
The greatest contribution to air pollution in Pristina	729	1456	375
PM_2.5_ emissions sources	1377	256	927
Ozone	389	1275	896
Vulnerable groups	1565	438	557
Vulnerability in general population	667	1353	540
Premature death due to air pollution	1511	434	615
Diseases caused by air pollution	2020	34	506
Effect of speed limits on PM emissions	1720	220	620
Staying inside during high levels of pollution	1423	524	613
Reliable air quality information	876	91	1593

**Table A2:** Responses to attitude questions.

Question	Do Not Agree at All	Do Not Agree	Not Sure	Agree	Completely Agree
Traffic jams can increase small inhalable dust locally	180	39	1025	813	503
People should pay more attention to how to reduce their individual contribution to air pollution	125	95	509	805	1026
Polluted air (fog/smog) bothers me, but I don’t think it poses a health risk *	380	450	433	637	660
Air pollution should be considered as a serious threat to the future development of society	192	179	499	855	835

* Coded in reverse order: Completely agree/Agree/Not sure/Not agree/Not agree at all.

**Table A3:** Responses to perception questions.

Question A1–A4	Not at All	Not Dangerous		Not Sure	Dangerous	Very Dangerous	
Air pollution (AP) is dangerous to my health	56	65		301	758	1380	
AP caused by heating and cooking is dangerous #	183	439		840	573	521	
AP caused by industry is dangerous	102	174		708	977	599	
AP caused by transport is dangerous	91	143		774	1067	485	
**Question A5, A7**	Never	Once a month or less		Several times a month	Once a week	Several times a week	Every day
How often do you notice AP	171	871		782	276	295	165
Have you had difficulty breathing last month	253	168		96	843	840	360
**Question A6**	Not at all		A little		Somewhat	A lot	
How concerned are you about AP in your area	1026		1255		140	139	

# 4 answers missing.

**Table A4:** Responses to behavior questions.

Question (Last Month)	Never	Rare	Sometimes	Always
Use public transport, walk, cycle	388	653	848	671
Turned off light, AC, etc. #	218	581	1035	725
Reduced outdoor activities because of AP	325	800	903	532

# 1 answer missing.
